# Visualization of Interstitial Pore Fluid Flow

**DOI:** 10.3390/jimaging8020032

**Published:** 2022-01-30

**Authors:** Linzhu Li, Magued Iskander

**Affiliations:** Civil and Urban Engineering Department, Tandon School of Engineering, New York University, 6 Metrotech Center, Brooklyn, NY 11201, USA; LL3256@nyu.edu

**Keywords:** roundness, granulometry, shape, inter-particle, microscale

## Abstract

Pore scale analysis of flow through porous media is of interest because it is essential for understanding internal erosion and piping, among other applications. Past studies have mainly focused on exploring macroscopic flow to infer microscopic phenomena. An innovative method is introduced in this study which permits visualization of interstitial fluid flow through the pores of a saturated synthetic transparent granular medium at the microscale. Several representative images of Ottawa sand were obtained using dynamic image analysis (DIA), for comparison with flow through perfect cylinders. Magnified transparent soil particles made of hydrogel were cast in 3D printed molds. Custom 3D printed jigs were employed for accurate positioning of the particles to ensure that particles have the same flow area within the soil. The pore fluid was embedded with silver-coated hollow microspheres that allowed for their florescence and tracking their movement within the model when illuminated by a laser light source. Images of the flow were captured from the model using a high-speed camera. This, along with particle image velocimetry (PIV) provided for the velocity and direction analysis of fluid flow movements within the pore space of a planar 2D model. Comparison of interstitial flow through homogeneous porosity-controlled Ottawa-shaped and cylindrical particles demonstrates that the magnitude of turbulence is related to particle roundness.

## 1. Introduction

Fluid motion through porous media is an important problem of interest to many fields of science and engineering. In geotechnical engineering, applications such as seepage induced instability, internal erosion, piping, and earth dam design are fundamentally flow problems. Similarly, problems where rapid generation and dissipation of pore water pressures are significant, such as liquefaction and cavitation, all benefit from advances in this area. Flow through the pores of a granular media can be studied at the macro scale or at the micro/pore scale. Empirical experimental studies often rely on macro scale measurements that focus on global parameters such as overall pressure drop which are typically used to estimate the behavior of porous media. In addition, filter design to prevent erosion or clogging has conventionally been studied using these empirical approaches. However, the fundamental processes that lead to the internal erosion in embankment dams and settlement due to migration of fines initiated at the particle scale remain elusive. Therefore, a basic understanding of fluid flow at the pore scale is critical for advancing knowledge impacting a wide range of practical geotechnical problems. 

Particle size has long been considered as the primary factor affecting flow in soils [1,2,3]. However, although particle shape is increasingly being recognized as having a significant influence on strength and deformation properties of sand (e.g., [4,5,6]), its effect on flow properties has only been recently explored (e.g., [7,8,9,10]). Particle shape determines the size and geometry of the pore space, which can be directly related to flow and capillary rise [11]. In addition, surface shape and roughness of the particles may contribute to the reduction of pore space where free-water flow may occur. Thus, particle shape characteristics determine the effective porosity that controls the soil’s hydraulic conductivity [12]. The available studies focused on the influence of the pore size distribution or particle shapes on the global flow properties with, none of the available studies providing an avenue for exploring the underlying flow regimes affecting the global flow properties. 

Generally, flow computations in porous media employ lumped parameters for hydrodynamic calculations. Indeed, while this approach provides reasonable estimates of flow quantities, it may not fully account for the entire complex flow structures that exist within the soil bed. In recent years researchers have explored the role of particle shape on flow using numerical simulations employing the discrete element method (e.g., [13,14]). These studies confirm long standing hypothesis but do not reveal new phenomena. A few experimental studies have employed particles with controlled geometries (e.g., [15,16]) However natural particles exhibit complex sphericity, roundness, and convexity [17,18] that cannot be represented by simplified controlled geometries. 

The present experimental study presents a methodology that permits visualization of complex flow paths within the soil using a combination of (1) transparent soils, (2) accurate acquisition of particle shapes using dynamic image analysis, (3) scaled up soils, (4) nano sized tracer particles, (5) laser illumination, (6) high speed imagery, and (7) using particle image velocimetry technique (PIV). 3D printing was employed for fabrication of custom molds to manufacture scaled up surrogate particles as well as specialized filters to ensure uniform flow. Experiments were performed on two particle systems with pore-controlled circular and Ottawa Sand, which enabled precise definition of shape characteristics and the determination of its influence on interstitial flow patterns. Velocity field measurements were obtained using PIV which permitted delineating the complex flow structure. Quantified analysis on the fluid flow within the void space of sands demonstrates that particle shape influences flow characteristics within pores of granular media which can potentially provide valuable information regarding flow. 

The advent of high-fidelity imaging systems has made it possible to observe kinematics of granular media at unprecedented micro scales. These methods involve expensive equipment and limited resolution [19]. 

## 2. Imaging of Macroscopic Fluid Flow

Bench scale model tests are frequently employed to study a variety of flow problems in porous media. The main purpose of model tests is to infer new phenomena, calibrate numerical models, or devise remediation plans [20]. Determining conditions within models by sampling from inside the model is intrusive, likely impractical, and introduces compliance errors. Therefore, a number of non-intrusive imaging techniques are increasingly employed to probe the interior of bench scale models involving porous media. Research manuscripts reporting large datasets that are deposited in a publicly available database should specify where the data have been deposited and provide the relevant accession numbers. If the accession numbers have not yet been obtained at the time of submission, please state that they will be provided during review. They must be provided prior to publication.

### 2.1. Measurments in Opaque Media

Most prior application of visualizing flow dealt with macroscopic flow. In geotechnical engineering, early studies dealt with visualizing the flow against a transparent wall to map the concentration of Nonaqueous Phase Liquid (NAPL) flow. Typically, two-dimensional transparent chambers containing either natural sand or glass beads were employed (e.g., [21,22,23,24]). In these studies, color tracers are employed to track the NAPL migration at the model boundaries with red and blue being the most common tracers (e.g., [25,26]). In a few cases, fluorescent tracers were also employed to profile NAPL concentrations (e.g., [27]). Typically, pixel information is transformed into NAPL concentration, with grayscale analysis being most common, except for a few studies that employed color formats to quantify the spatial saturations of NAPL (e.g., [28]). Although 2D modeling provides valuable insight into contaminant transport, it presents difficulties when results are extended to 3D systems. Therefore, there is a need for three-dimensional laboratory models that can be used to track migration of contaminants over time. 

Visualization of three-dimensional flow in natural media can be carried out using a variety of non-destructive testing methods including resistivity [29], X-ray tomography [30], X-ray micro tomography [31], and magnetic resonance imaging [32]. These methods have been adapted to visualize NAPL transport in porous media, with different spatial, temporal, and spectral resolutions [33,34,35]. However, these methods are not only expensive but require considerable expertise in their application which limits their widespread use. In addition, use of electromagnetic energies require a significant trade-off between temporal and spatial resolutions [19]. On the other hand, optical imaging techniques are non-intrusive, relatively inexpensive, expedient, and simple enough for use even in K-12 laboratories [36].

Glass beads have been extensively used in research of 2D flow problems (e.g., [37,38,39,40,41,42]). However, glass beads are translucent, which limits the ability to visualize flow conditions within the model. Thus, glass beads have been primarily used due to the uniformity of their physical properties rather than their optical properties. 

### 2.2. Measurments in Transparent Soils

Transparent synthetic soil surrogates representing a wide range of natural materials have been developed [43]. These synthetic surrogates are made by matching the refractive index of solid particles and a pore fluid to minimize refraction of light, which is a major source of transparency degradation [44]. Several families representing the macroscopic behavior of sands and clays have been developed, but each family possesses a different refractive index [45], so mixing the materials is not straight forward, but has nevertheless been achieved with success [46]. Both Silica Gel and Fused quartz have been employed for representing sand [47,48]. Precipitated Silica, and Laponite have been used to model stiff and soft clays, respectively [49,50,51,52]. Aquabeads has been used to model a wide range of grain sizes [53]. A variety of pore fluids can be used including mineral oil blends, pre-manufactured sucrose solutions and sodium thiosulfate-treated sodium-iodide (STSI) [54,55,56,57]. To date transparent soils have been used to investigate a variety of soil structure interaction and flow problems [58]. 

The introduction of transparent soils permitted visualizing flow conditions within the model. Early applications involved simulation of multiphase flow and surfactant flushing in layered 2D synthetic soil surrogates [59,60]. The methodology involved correlation of image luminance (grayscale) with contaminant concentration at the boundary of 2D models. Fenandez Serrano et al. [61] extended the system by employing 3 cameras to track subsurface point source contamination transport of NAPLs in 3D. The optical model to determine the 3D concentration of contamination within a soil medium was shown to be feasible, inexpensive, and non-invasive; for modeling a specific soil formation having a pre-determined grain size distribution and saturation profile. 

Kashuk et al. [62] observed that chromatic families of color space are less sensitive to optical noise, compared to luminance (grayscale) components. This was used to reconstruct contamination plumes in 3D transparent soil models. First, correlation between pixel information and color dye concentration in different color spaces was exploited to identify the optimum color for analysis. A color space is a numerical way to define individual colors and using color space is one of the main approaches for expanding the information deduced from pixel information [63]. Typically, color spaces consist of three- or four-color components. These components can be mathematically combined to reproduce each unique color within an image. Using common commercial digital cameras, the image pixel information is originally stored as RGB color space data. This space consists of three components: red, green, and blue. However, RGB data can be transformed into a variety of different color spaces [64]. Correlation analysis was carried out for several dyes and 24 color spaces to identify the best color dye tracer and its concentration as well as the ideal color space components for rendering a dyed dense NAPL (DNAPL) contamination within a transparent soil aquifer [65]. Second, the concept of integrated concentration and peak signal to noise ratio were combined to relate color intensity to the extent of contamination [66]. Third, a novel iterative reconstruction algorithm named 3D carving was also used to resolve three 2D projections into a 3D model [67]. The methodology successfully estimated the volume of injected and recovered dyed DNAPL and reconstructed the 3D distribution of contamination inside the transparent soil model. A resolution of 1,000,000 voxels was achieved, with errors estimated as ~8% of the DNAPL volume, which corresponds to 2% of the total reconstructed voxels [68]. 

Visualization of flow within the soil pores remains in an early stage of development. Early studies involved the use of tracers (e.g., [69]). Visualization of flow within interstitial pores was first demonstrated by Hassan and Dominguez-Ontiveros [70] in materials with simplified geometries in order to visualize flow in gas-cooled pebble bed reactors for nuclear power generation. The technique has also been recently adapted by Khayamyan et al. [15] to demonstrate turbulent flow in a bed of sphere using stereo PIV. In geotechnical engineering, the methodology has been extended by Hunter and Bowman [71] to explore internal erosion in gap-graded particulate systems. Recently, Sanvitale et al. [72] combined particle-scale 2D experiments using transparent soils and 3D computational fluid dynamics to reconstruct 3D pore flow-fields. 

## 3. Imaging of Inter-Particle Fluid Flow

### 3.1. Experimental Methods

**Figure 1:**
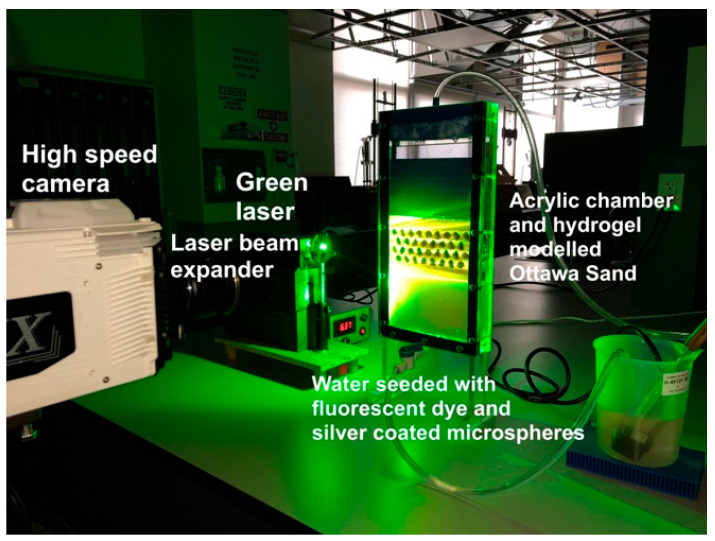
Test setup used to visualize fluid flow through pores of a granular material.

This study focuses on imaging of interparticle flow. The process requires the use of a high resolution and high-speed camera. Equipment to do so is indeed available, but very costly. The available camera is only capable of capturing up to 5 Mega-Pixel images at a frame rate of 250 Hz. Thus, the authors opted to scale up the particles in order to overcome the limitation of the resolution. Flow within the interstitial space was imaged by tracking the motion of fluorescent micron-size tracer particles that were made to fluoresce when illuminated by laser light. Refractive index matched materials to produce transparent granular media were used in order to permit laser light penetration into the model. Transparent soils have been used for over 25 years to permit visualizing conditions within soils [45]. In addition, only one high-speed camera and laser are available, so the problem was studied in 2D. Thus, flow fields within the pores between scaled particles were analyzed by Particle Image Velocimetry (PIV). PIV is a pattern matching algorithm used extensively for motion estimation in fluid dynamics [73]. A time-resolved displacement field was obtained from flow through the interstices of the granular media by performing PIV on consecutive images obtained from the high-speed camera. The methodology is demonstrated in a YouTube video depicting high-resolution flow of a different experiment projected in slow motion [74,75]. The following are the details of the system components (Figure 1). 

### 3.2. Scaled-Up Synthetic Granular Particles

Scaled up soil grains were made of Polyacrylamide hydrogel. Polyacrylamide was selected due to it having a refractive index (*RI*) close to that of water (*RI* = 1.33). The material was acquired from Bio-Rad laboratories and was fabricated in-house following procedures similar to those described by Byron et al. [76]. The hydrogel concentration was chosen by trial and error, on account of its stiffness and refractive index. The selected hydrogel concentration of 12% was determined as suitable for the current test, mainly due to its *RI* matching with that of water, and for its sufficient stiffness when subjected to flow under a range of hydraulic gradients. Lower concentrations of Polyacrylamide resulted in transparent particles that were not sufficiently stiff for accurate placement within the model, while higher concentrations were not sufficiently transparent to permit laser penetration through the entire width of the model. 

**Figure 2:**
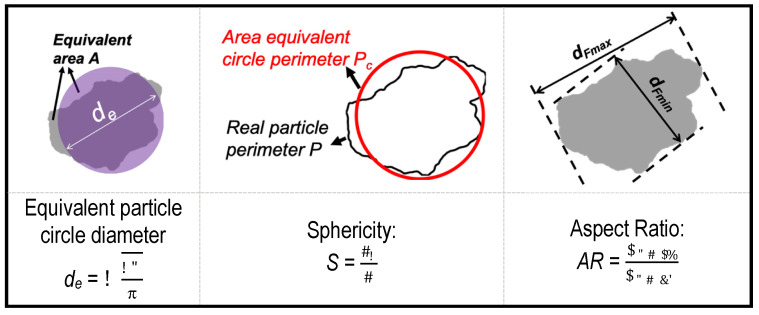
Graphical explanation of particle size and shape descriptors.

**Figure 3:**
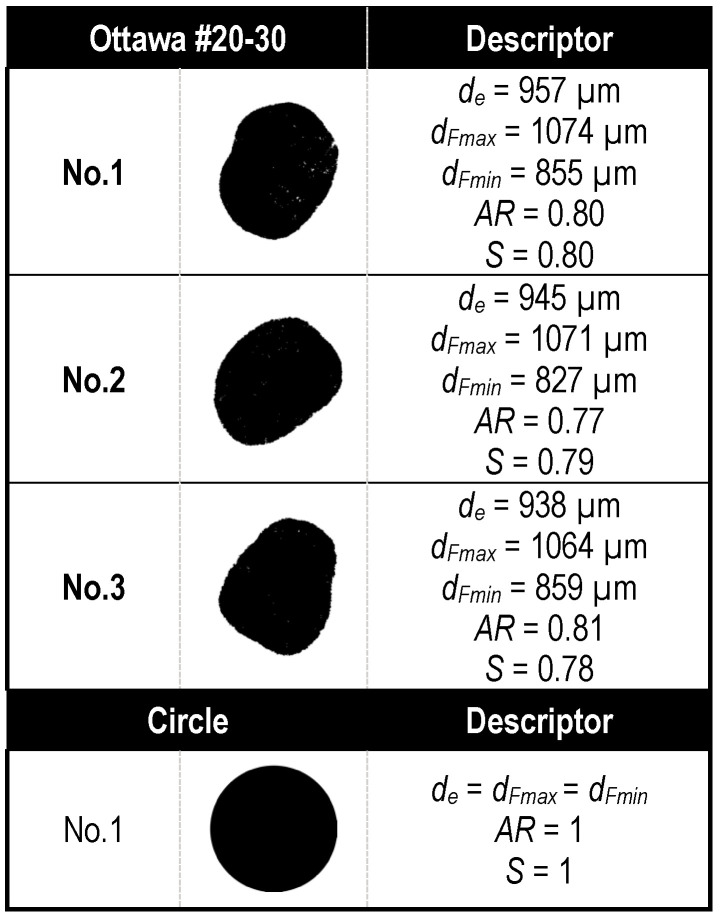
Images of Ottawa #20-30 sand particles captured using Dynamic Image Analysis (**Top**) compared to synthetic circular particle (**bottom**).

Flow in natural Ottawa sand #20-30 was compared to flow between perfect spherical particles. The exterior shape of the Ottawa sand particles was obtained using Dynamic Image Analysis [77]. Dynamic image analysis is a powerful particle size and shape analysis tool commonly used in pharmaceutical and powder technology applications. The method relies on a pulsed light source and a high-speed camera to capture the size and shape of particles. A commercial dynamic image analyzer, *QicPic*, was used in this study to capture particle projections at a frequency of 500 frames per second, and to generate high-resolution particle images of particles in the size range of 4–2888 μm. A typical analysis provides single particle image projections and particle size distribution along with several size and shape descriptors (Figure 2). Representative particle images and size information of Ottawa sand #20-30 used in this study are shown in Figure 3. Details of operation and analysis procedures for accurately capturing representative 2D projections of natural sand particles using DIA are described by Li and Iskander [78]. 

The authors selected 3 individual particles of Ottawa sand such that they had different shapes but a similar Equivalent Particle Diameter (EQPC) of 947 ± 10 µm (Figure 3). These values were scaled by a factor of ~15 such that the test particles were approximately 14 mm in EQPC. The magnification was chosen to accommodate manufacturing of the hydrogel particles, and also to allow for adequate resolution for imaging of interstitial fluid flow. 

**Figure 4:**
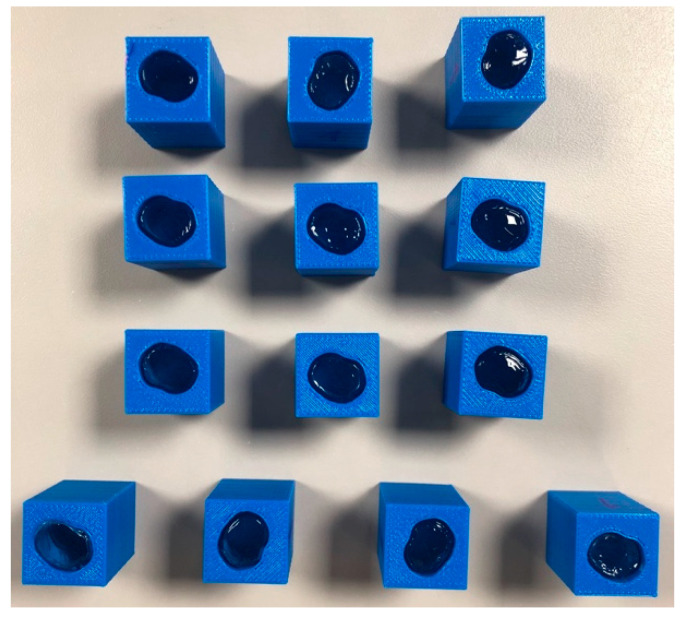
3D printed molds for casting enlarged extruded Ottawa #20-30 particles. Note Hydrogel modelled particles cast in the molds.

The 2D DIA images were extruded in Fusion 360 software and subtracted from solid volumes in order to produce molds for making hydrogel particles. At the same time cylindrical molds having a diameter of 15 mm were also produced. All molds had a thickness of 25 mm. Forty 3D printed molds were manufactured in house for casting each of the round and Ottawa shaped hydrogel particles (Figure 4). The molds were coated with mineral oil to help with extracting the cured hydrogel particles from inside them. 

Hydrogel solution was made by mixing 100 mL of 12% Polyacrylamide (PAC) with 40 mL of 30% acrylamide solution and 59.4 mL of Deionized water. 0.5 mL 10% aqueous ammonium persulfate (APS) and 0.1 mL catalyst Tetramethylethylenediamine (TEMED) were then added to the solution. The mixing process did not result in air entrapment, thus placement under vacuum was not necessary. The resulting particles were sufficiently clear to permit the laser light sheet to penetrate through the entire model width. After mixing, the solution was poured into the mineral oil-coated 3D printed molds (Figure 4). The particles cured after 2–3 h and became stiff in the molds. Cured hydrogel particles were removed and stored in de-ionized water for 24 h, during which they swelled by absorbing water. It was observed that the tested hydrogel particles expanded by approximately 10% in length dimension compared to the mold size and little expansion was observed beyond that point, as also reported by Byron et al. [76]. Additional expansion of hydrogel particles can be problematic if the test is not run expeditiously because expanded particles do not fit in the precision-fit mold. Particles were preserved in a refrigerator to prevent microbial growth. The resulting particles resembled extruded two-dimensional Ottawa sand and cylindrical sand, which were then stacked randomly in a planar chamber to permit study of 2D flow between particles having different shapes. 

Hydrogel particles have a *RI* 1.38 at a concentration of 12% Polyacrylamide measured at 23 °C. This value is close to the *RI* of the pore fluid (*RI* water = 1.33), which permitted both (1) transparency of the saturated 2D particle assembly and (2) identifying the outline of the particles easily with the naked eye. Water is preferred over commonly used refractive index-matching fluids in conducting seepage tests, not only because it is less hazardous compared to other fluids but also the complications resulting from viscosity scaling are largely eliminated.

### 3.3. Testing Apparatus

**Figure 5:**
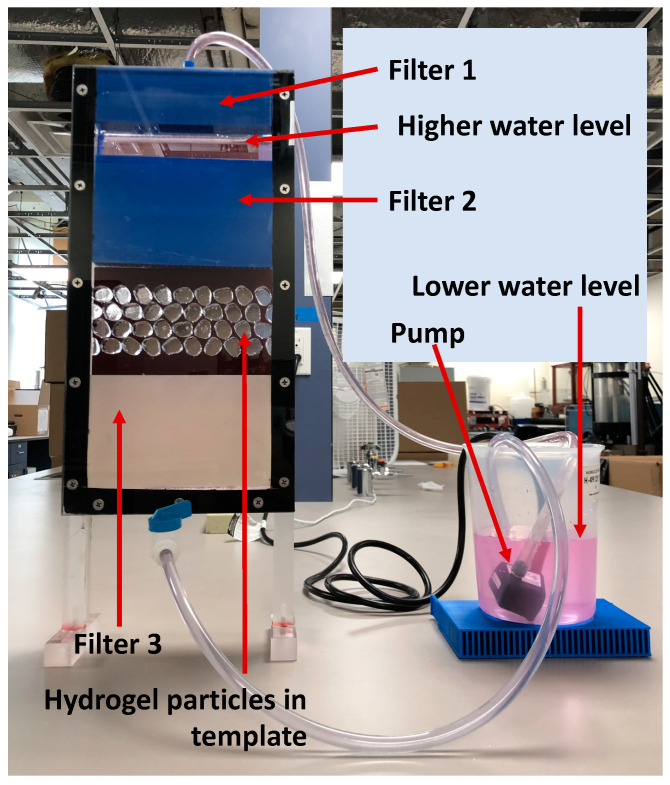
Testing apparatus showing hydrogel particles placed at a fixed porosity and saturating water infused with fluorescent nano particles.

**Figure 6:**
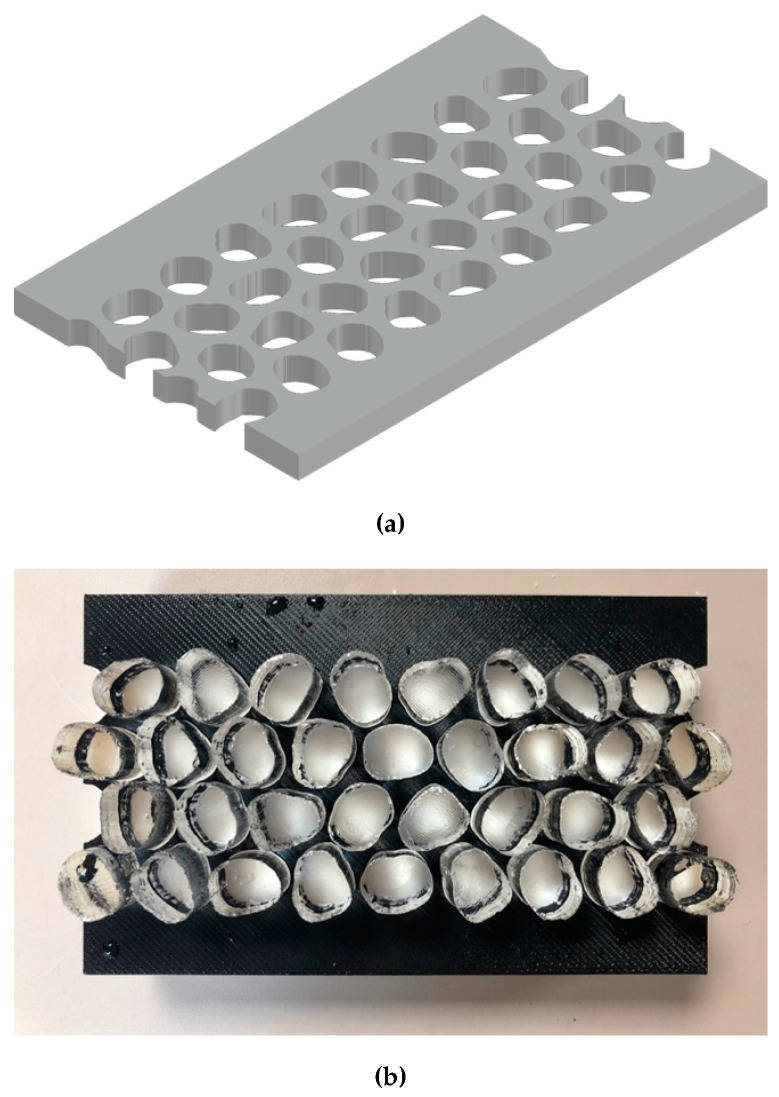
Custom template employed for precise distribution of particles within the model. (**a**) 3D printed template (top), and (**b**) template with hydrogel particles placed within it (bottom).

A constant head planar permeameter was designed for visualization of fluid flow through the interstitial pores of the granular media. The setup design was inspired by ASTM D2434 [79], with downward seepage flow. The setup consisted of a cuboid planar permeameter and a water reservoir (Figure 5). The internal dimensions of the permeameter were 200 mm by 25.4 mm by 360 mm (length × width × height). The dimensions of the chamber were selected to permit placement of 8 to 10 particles across the permeameter, depending on the test. The particles were typically 15 mm in diameter, and that size was selected to allow for the imaging system to capture flow in the interstices of the granular media. This width meets the specifications of [79], which requires that the chamber be 8–12 particle diameters in size. Particles were typically placed as 3 or 4 rows and held in place inside a template matching their exact perimeters. The use of a template was necessary to ensure that the interstitial flow pathways met strict design requirements, averaging 3 mm wide. The width of the template was 8 mm leaving a flow path that was 17 mm thick (Figure 6). All particles were placed such that their centers were spaced 18 mm from each other, leaving a nominal interstitial flow path of 3 mm. Cylindrical particles were evenly spaced, while Ottawa particles were randomly placed. Random placement of the hydrogel in water is believed to permit simulating the morphology of water deposited sand formations, while even placement of cylindrical particles provides for a controlled experiment for comparison. Initially, some sidewall leakage was observed but it was easily eliminated by adjusting the width of the chamber to accommodate particle shrinkage. 

Uniform placement of the particles in the mold with the aid of a template was able to overcome some previously encountered experimental difficulties. Li et al. [80] observed that random placement of 2D particles in a planner mold contributed to the formation of closed and connected pores. Closed pores exhibited regions of high vorticity and small-scale turbulence; which are believed to be an artifact of 3D effects occurring in the 2D setup. Connected channels accounted for the majority of the flow volume, but high flow speeds were associated with narrow channels between some particles. Both experimental artifacts were eliminated from the current setup by uniform placement of the particles. 

**Figure 7:**
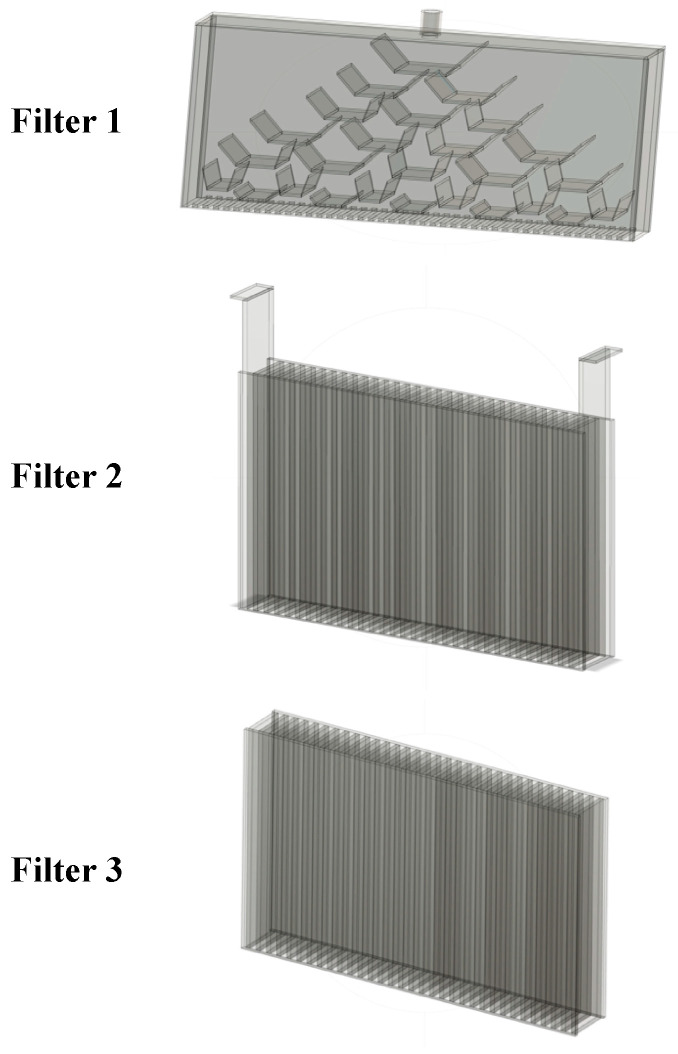
CAD models of custom 3D printed filters employed for distribution of flow within the model (corresponding to filters shown in Figure 5).

Three custom 3D printed filters were employed to evenly distribute the water flow above and below the observation window (Figure 5). The authors experimented with many designs and the design shown in Figure 7 was the most successful in largely achieving uniform vertical flow. In our experience it was necessary to place filters at the top and bottom of the observation window to achieved uniform flow. It is noteworthy that the filters above and below the observation window have openings that are 2 mm wide, while the interstitial spaces between the particles were larger than 3 mm wide. Thus, the flow rate and seepage velocity were influenced by the choice of filters 2 and 3, but the same filters were used for all tests reported in these studies. 

The permeameter accommodated imaging flow under both transient and steady state conditions. Steady state flow was investigated in this study under constant head conditions. An adjustable water pump was used to circulate water through the sample. The elevation of the water reservoir and pump power were adjusted to maintain a constant head and change the hydraulic gradient during different tests. Hydraulic gradients of 5.7 and 4.6 was used for Ottawa sand and Cylindrical sand, which corresponds to a discharge velocity of 0.058 and 0.024 cm/s. Different hydraulic gradients were necessary to accommodate the capacity of the available circulating pump. 

### 3.4. Fluorescent Tracer Particles

Fluorescent particles have been a staple of fluid dynamics for several decades. Particles are typically seeded with the flow stream in order to track motion of gases and liquids. The technology has been adapted in this work to trace the motion of pore fluid within simulated soil voids. Reflective coated microspheres were added to the fluid to improve the fluid contrast required for PIV analysis. Approximately 1 g of tracer particles was mixed in the water tank. The microspheres employed were made of silver-coated hollow glass spheres having a bulk density of 1.6 g/cc. They have an average diameter of 13 µm (CONDUCT-O-FILR^®^ Product ID: SH400S20). The material was sourced from Potters Industries^®^ (Malvern, PA, USA). A 13 µm size tracer particle was selected in order to eliminate compliance errors since their size is considerably smaller than the average flow channel (~3000 µm). The microspheres followed the fluid motion and, as a result, their motion captured during the test yielded the motion of the fluid within the interstices of the granular media. 

### 3.5. Optical Setup

The optical setup was designed to visualize a plane within the permeameter during steady state flow through the granular media to allow for imaging of the seeding microspheres. Since the hydrogel and the pore fluid had comparable *RI*, the saturated granular media was transparent. In order to capture the flow field, the set up shown in Figure 1 was designed. 

A laser light source was used to illuminate a plane within the permeameter chamber. The laser was a diode-pumped solid-state green laser with a maximum output of 2 W. A beam expander and a magnifier lens were used to produce a planar laser sheet. 

A high-speed camera (NAC HX-5) was used to capture movements of the seeded plane during flow, at a frame rate of 250 frame/s. A Tamron 90-mm macro lens was attached to the high-speed camera. The lens was focused on the laser sheet. Exposure was adjusted using the lens aperture. No other illumination was permitted at the lab during testing. The interaction of the laser light and the tracer particles within the laser sheet plane scintillated the seeded microspheres, resulting in a speckle pattern that could be imaged during testing [75]. 

Uncompressed gray-scale Tiff images were captured after steady-state flow was established. Approximately 1000–1200 images were captured for each test. Images were stored in the camera memory until the tests was over, at which point they were transferred to a PC for further processing. 

Inspection of the captured images reveals that (1) irregular particle boundaries are clearly discernable due to the slight mismatch in the refractive indices between the pore fluid and the Polyacrylamide particles, and (2) that tracer particles effectively capture the flow trajectories throughout the observation area. 

### 3.6. Digital Image Correlation

PIV is a form of digital image correlation (DIC) that has been a staple of fluid dynamics measurements for over a decade. The technique is based on finding the shift between consecutive images that minimizes their differences. PIV employs a correlation function to locate the best matching position of two images, before and after deformation, to determine the average displacement between the two images. Images are divided into grids for this purpose, and the distribution of flow is obtained by identifying the relative displacements between corresponding windows. An optimization scheme is carried out to find the displacement field between two images before and after motion. For DIC, it is of paramount importance to select the optimized criterion for analysis [81]. We used the zero- mean normalized sum of squared difference (ZNSSD) approach; this method minimizes the sum of the difference between corresponding interrogation windows [82]. 

**Figure 8:**
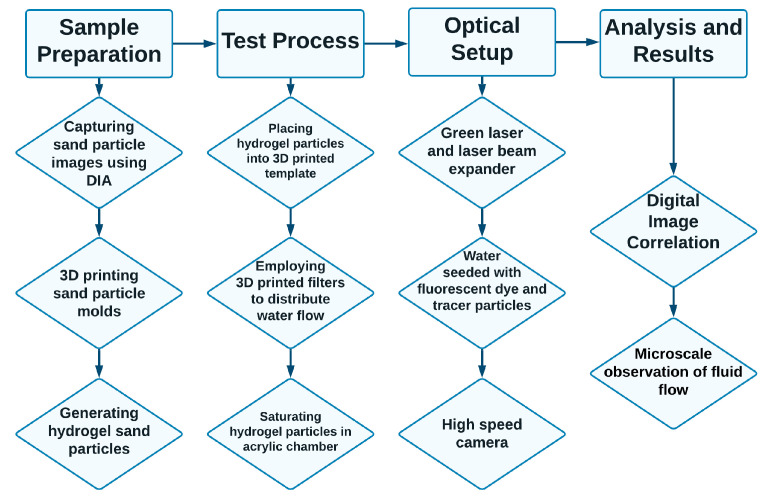
Overall procedures for visualization of interstitial pore fluid flow.

PIV was carried out by using PIVview 3C, which is a software package for analysis developed by PIVTEC in Germany. A window size of 32 × 32 pixels was employed. An overlap between the interrogation windows was chosen to be 50% of the final window size (16 pixels), which resulted in calculation of displacement fields on 32 × 32 pixels, which corresponds to a 1.3 × 1.3 mm grid. The analysis was performed in the frequency domain to speed up the computations. Features of the PIV algorithm used consisted of a multi-pass and multi-grid interrogation algorithm, as well as sub-pixel estimation, the details of which can be found in [83]. The experimental workflow is summarized in Figure 8. 

## 4. Microscale Observation of Fluid Flow 

**Figure 9:**
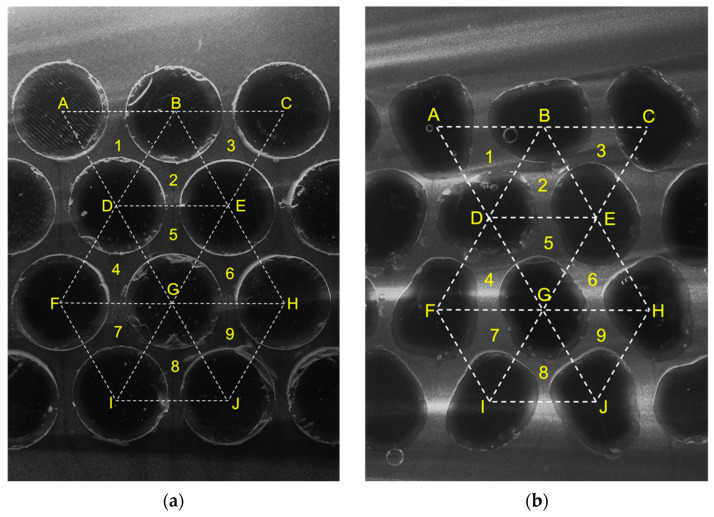
Typical flow images captured by the high-speed camera for (**a**) cylindrical particles and (**b**) simulated Ottawa sand. Note triangular zones used for analysis of flow superimposed over the captured images.

After steady state flow was achieved, images of flow were captured by the high-speed camera (Figure 9). Steady state was initially established based on visual observation of the upper and lower water reservoir levels (Figure 5), and later verified through PIV analyses. Although both models have a similar void ratio *e* = 0.59 different average flow velocities and hydraulic conductivities were observed. The observed hydraulic conductivities *k* for Ottawa and Cylindrical particles were 5.7 and 4.6 cm/s, respectively. These findings are consistent with the notion that particle shape affects hydraulic conductivity and are explored further in this section. 

### 4.1. PIV Analyses

Consecutive image pairs were analyzed using the PIV techniques described in the previous section. The images acquired during the test are presented in Figure 9. These images captured a scaled flow between magnified natural particles. The tracer particles between hydrogel particles can be seen in these high-resolution images. The boundaries of particles are discernable because of a certain degree of refractive index mismatch between water and Polyacrylamide particles. 

**Figure 10:**
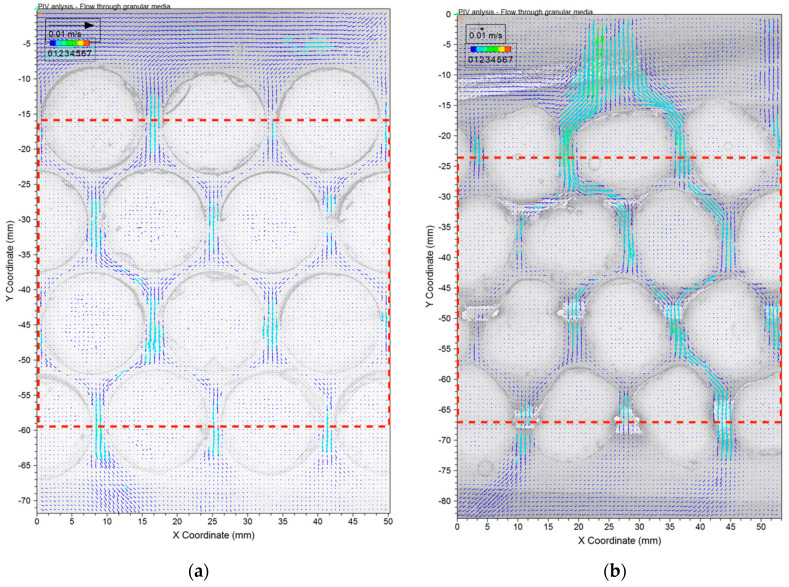
Flow images analyzed using Particle Image velocimetry (PIV) for (**a**) cylindrical particles and (**b**) simulated Ottawa sand.

Results of PIV analyses on images acquired at two consecutive times in Figure 9, are shown in Figure 10. Flow was easily identified inside the pores between hydrogel modelled sands. However, a threshold velocity was required in order to mathematically distinguish between solid particles and flow. A threshold of displacement corresponding to 0.1 pixel was employed which corresponds to a velocity of 0.01 cm/s. 

**Figure 11:**
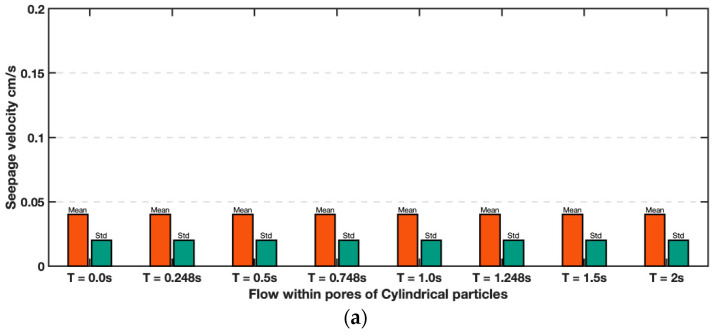

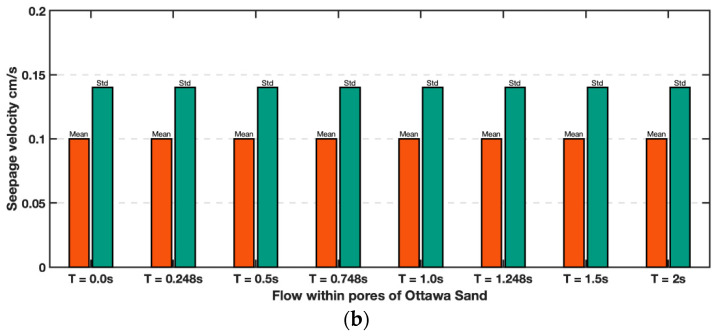
Comparison of mean interstitial flow velocities in 8 consecutive frames for flow between (**a**) Cylinders (top) and (**b**) simulated Ottawa sand particles (bottom).

The general pattern of flow within the porous media depended on the shapes of the modelled sands. Evenly distributed flow was better achieved at a slower discharge velocity *v* = 0.024 cm/s in cylindrical particles, while a faster velocity of *v* = 0.058 cm/s was required for the Ottawa particles. In any case, comparison of successive PIV analyses between 8 consecutive frames demonstrates that steady flow was indeed established, and the flow was uniform within the model for both materials (Figure 11). Note that although models exhibit different velocity, they are both uniform. It is however interesting note that the coefficient of variation (standard deviation normalized by the mean) is noticeably higher for the Ottawa sand than for the cylindrical particles. 

### 4.2. Discharge Velocities

**Figure 12:**
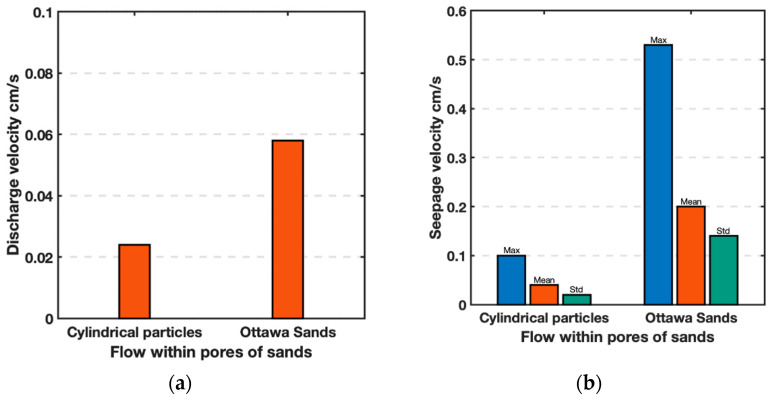
Comparison of average (**a**) discharge and (**b**) seepage velocities between perfect cylinders and simulated Ottawa sand particles.

Porosity was computed for the flow area under consideration ignoring the rest of the model using the red dotted boundaries shown on Figure 10a,b as the area of particles divided by the area of the dotted red square. It is noteworthy that both models have a porosity *n* = 0.37. Nevertheless, different hydraulic gradients were required to achieve for steady state. Theoretically, seepage velocity (*v_s_*) is equal to the discharge velocity normalized by the porosity, *v_s_* = *v*/*n*. The values of *v* for Ottawa and Cylindrical particles were measured in the experiment as 0.058 and 0.024 cm/s, respectively. Both values were obtained as the collected volume of water (*V*) normalized by the given time (*t*) and the cross-sectional area (*A*) of soil media, *v* = V/(t×A). Theoretically these values correspond to *v_s_* of 0.16 and 0.06 cm/s, respectively. However, the observed *v_s_* for Ottawa and Cylindrical particles are 0.2 and 0.04 cm/s, respectively (Figure 12). The values are close to the theoretical values, but one is higher while the other is lower. Clearly particle shape affects the seepage velocity as well as its relationship to the discharge velocity. 

### 4.3. Interstitial Seepage Velocities

**Figure 13:**
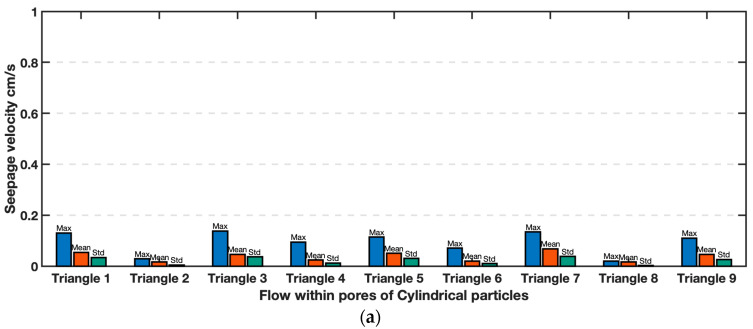

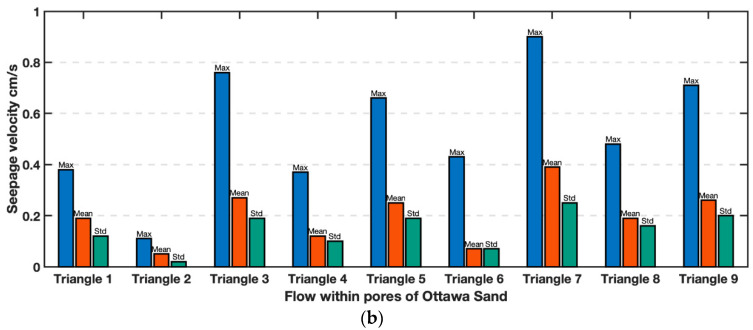
Distribution of flow velocities in between (**a**) Cylindrical particles (*T* = 2 s) and (**b**) simulated Ottawa sand particles (*T* = 0.248 s).

The spatial distribution of flow within each model was explored using statistical analysis of fluid flow velocity within each triangle shown in Figure 9, for each material. The maximum, mean and standard deviation of flow velocity within each observation triangle are presented in Figure 13. The vertex of each triangle is located at the center-of-area in each particle, thus, the area of pores between cylindrical and Ottawa sands in each triangle are equivalent. Nevertheless, it can be seen that the seepage velocity in each pore (i.e., triangle) is somewhat different, even for the cylindrical particles that possess a uniform geometry. The maximum and standard deviation of velocity in Ottawa Sand exhibit a larger difference in each pore compared to cylindrical particles. These differences are consistent across many analysis frames as seen previously in Figure 11. Variations in seepage velocity reflect turbulence which likely resulted from the effects of irregular particle shape in Ottawa Sand, relative to cylindrical particles. 

**Figure 14:**
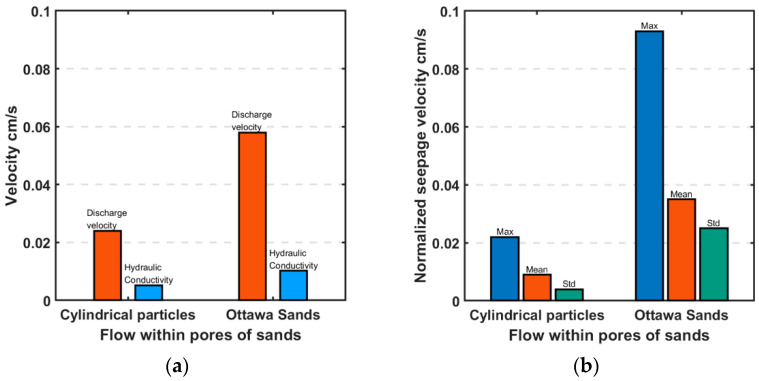
Comparison of hydraulic conductivities (velocity v, normalized by hydraulic gradient, i) between Cylindrical particles and simulated Ottawa sand particles. (**a**) Discharge velocity, and (**b**) Seepage velocity.

The average values of fluid flow for all 9 triangular windows shown in Figure 13 represent seepage velocity. The seepage velocities of Ottawa and cylindrical particles are compared in Figure 14. As expected, a smaller maximum and mean fluid velocity were observed in cylindrical particles compared to Ottawa sand, due to different discharge velocities, however, these values were approximately 2–3 times larger than the discharge velocity for each test. Discharge velocity may have been influenced by the overall experimental set up such as employed filters. Nevertheless, it is evident that flow within Ottawa sand experiences significantly more turbulence as evidenced by the standard deviation of fluid velocity in Ottawa sand is larger than the cylindrical particles.

### 4.4. Hydraulic Conductivities

Hydraulic gradient *i* is not linearly proportional to the discharge velocity (*v*) which contributed to a difference in the observed hydraulic conductivities (*k*). The observed *k* of Ottawa and Cylindrical particles were 0.0102 and 0.0052 cm/s, respectively. These values can be compared to the flow velocity normalized by hydraulic gradient, *v*/*i* or *v_s_*/*i*, and explored in Figure 14. Although both models have identical porosities and employ the same fluid, it is indisputable that particle shape affect the *k*, *v*, as well as the *v_s_*. This points clearly to the fact that interstitial flow is turbulent, even though Ottawa sand is relatively uniform in its shape. Angular sands are expected to experience far more turbulence. 

### 4.5. Flow Trajectories

**Figure 15:**
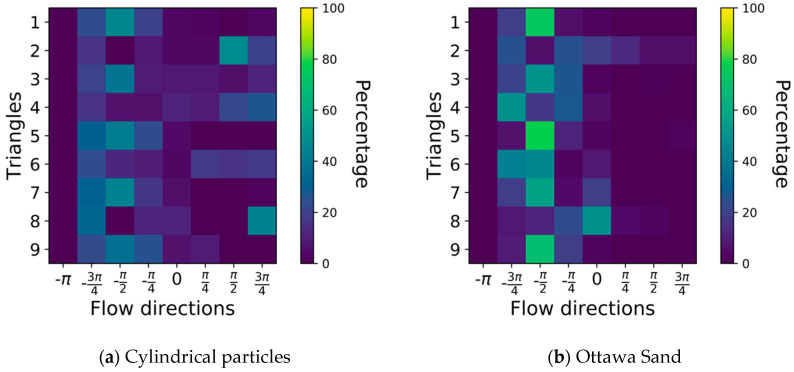
Comparison of the orientation of flow trajectories between (**a**) cylindrical particles and (**b**) simulated Ottawa sand particles.

Turbulence within the flow is further explored by investigating the orientations of flow trajectories within each observation triangle for both cylindrical and simulated Ottawa sand particles. The flow direction was calculated and presented using a histogram of oriented gradients (HOG). First the orientation, *θ*, of each velocity vector within an observation triangle is determined using Equation (1).

(1) $$\theta =\tan^{-1}\frac{\Delta y}{\Delta x},$$

where Δy and Δx are the vertical and horizontal components of the flow velocity. To facilitate comparison, orientations were grouped into 8 bins representing the directions of a circle such that each bin represents 45° as follows: $-\pi ,\frac{-3\pi }{4},\;\frac{-\pi }{2},\;\frac{-\pi }{4},\;\frac{\pi }{4},\;\frac{\pi }{2},\;\frac{3\pi }{4},\;\pi$. The HOG of interstitial flow between cylindrical particles is compared to the HOG of flow between extruded Ottawa particles in Figure 15. Each bin represents orientations at the bins indicated value of *θ* ± 22.5°. Note that $\frac{-\pi }{2}$ and $\frac{\pi }{2}$ represent downward and upward vertical flow, while π and −π represent horizontal flow towards the right and left sides of the observation window, respectively. 

For each observation triangle, an 8 bin HOG is employed to represent all possible flow orientations and their percentage of occurrence in that triangle, for a total of 72 bins representing the spatial distribution of orientations within the flow (9 triangles × 8 bins). At each triangle a heat map is used for displaying the percentage of flow that falls within each HOG bin. Several observations are possible. First, because the flow is downward, orientations within the 0–π rage are limited, but not zero. In fact, 10–20% of the flow exhibited upward trajectory in Ottawa Sand, while 20–30% of the flow trajectories exhibited upward flow in Cylindrical particles. These results are difficult to see in the PIV plots (Figure 10), which suggests that they were dominated by vectors with small magnitude. Together, these observations suggest that the smooth curvature of cylindrical particles and symmetrical geometry is prone to causing small turbulence, while turbulence between Ottawa particles is larger in magnitude. The second observation is that flow between Ottawa sand is more concentrated in the downward vertical direction than for the cylindrical particles. This is also visible in the PIV results (Figure 10). Note that even though all observation triangles have identical porosities, the cylindrical particles are perfectly placed, resulting in identical interstitial flow paths while the Ottawa particles are randomly spaced resulting in somewhat arbitrarily sized interstitial flow paths. Indeed, water follows the path of least resistance and small differences in the flow channels resulted in a more concentrated downward flow in the Ottawa particles than for the cylindrical ones, similar to the Mathew effect. 

### 4.6. Measured v. Predicted Interstitial Velocity

Kozeny–Carman equation (Equation (2)) provides an alternative definition of permeability based on geometric variables, including the hydraulic diameter (*D_h_* = 4*n*/*S_s_*), where *S_s_* = Specific surface area and the hydraulic tortuosity (*T*) which is defined as the ratio of the porous medium (*L*) length to the actual pore flow path (*L_e_*): *T* = *L*/*L_e_*.

(2) $$v=-\frac{n}{\mu }\left(\frac{D_{h}{}^{2}}{16C_{k}}\right)\left(\frac{\Delta P}{L}\right){\left(\frac{L}{L_{e}}\right)}^{2}=nv^{*}T^{2}$$

where,

$$T^{2}={\left(\frac{L}{L_{e}}\right)}^{2}=\frac{v}{nV}\;k_{c}=\frac{n^{3}}{S_{s}{}^{2}}\left(\frac{T^{2}}{C_{k}}\right)\;D_{h}=\frac{4n}{S_{s}}=\frac{4D_{m}n}{C_{s}\left(1-n\right)}=4R_{h}$$

However, in reality, hydraulic tortuosity cannot be precisely determined due to the extremely complex and invisible microscale flow paths encountered in real flow. A recent study [84] presented a technique that permits estimating the hydraulic tortuosity and the Kozeny constant at the microscale, using the *Superficial Effective Diameter* (*De*). *De* is defined based on the superficial velocity (seepage velocity *v_s_*) as “*another type of an effective diameter with a cylindrical friction constant and the same pressure drop but along the medium length (L).*” Kozeny’s tortuosity definition is the correlation between the apparent flow velocity (same as discharge velocity *v* in this study) and the interstitial velocity *v_i_* = *v*/*nT*. Accordingly, Reference [84] redefine the interstitial velocity term in Kozeny’s equation as a function of the actual flow path length (*L_e_*), and additionally defined the superficial effective diameter based on the superficial velocity (*v_s_*), and the effective tortuosity coupled with all determined variables such as porosity and hydraulic diameter. Reference [84] examined the influence of tortuosity in 3D porous media models exhibiting a wide range of porosity (13.4–47.4%) and permeability (0.0073–18.3 Darcy) to assess the effect of varying flow features in relation to the direct flow path. Depending on factors such as grain shape, arrangement, homogeneity, and path structure, tortuosity values were estimated according to [84] as *T* ≈ 0.63 and 0.88 for Cylindrical particles and Ottawa Sand, respectively. In this study, the interstitial velocities obtained from PIV are 0.10 cm/s and 0.19 cm/s, respectively (Table 1). Although the Interstitial velocity calculated using PIV for Cylindrical particles differs by −0.06 cm/s from the calculated value, the result for Ottawa sand is quite close to the calculated value (0.19 vs. 0.20 cm/s). These differences could be caused by 3D numerical modeling overlooking the complexity of the natural flow path or differences between 2D and 3D flow. Nevertheless, the present study demonstrates that the proposed methodology permits capturing complex interstitial pore fluid flow and provides a check on inferences derived from numerical simulations.

## 5. Practical Implications 

The use of 2D model particles to represent 3D natural particles was necessary to capture flow phenomena that otherwise have been indistinguishable. In micro-fluidics, the size effects are quite important owing to the combination of Fick’s and Knudsen’s diffusions, particle wall roughness among other factors [85]. 2D PIV method was applied in this study which required the adoption of the up-scaling concept. This of course introduces some errors in the similitude between the model and prototype flow. Thus, simple similarity theory could not be directly applicable for micro pore flows. In any case these errors are small in comparison to representing 3D flow as 2D flow. Nevertheless, this study demonstrates that 2D upscaled PIV method can be consistent with the real pore flows. However, the hydraulic conductivity of natural soils is often proportional to the square of the diameter of the particle size *D*_10_. Thus, although (1) water was used as a pore fluid and (2) the shape of the particles is similar to that of natural Ottawa sand, the up-scaled dimensions of the particles and flow channels correspond to a hydraulic conductivity that is larger than that of natural sands. This indeed may affect the magnitude of turbulence. Nevertheless, the authors believe that the phenomena observed at the magnified scale likely occur at smaller scales as well. In addition, comparison between interstitial flow between Cylindrical and simulated Ottawa sand suggests that the magnitude of turbulence is related to particle roundness. The observed turbulence exists within interstitial flow, that is rarely considered in practice. Thus, these observations may have profound implications for a variety of geotechnical, geo-environmental, and industrial applications such as filtration, environmental remediation, and resource recovery. 

In this work, PIV was used to visualize interstitial flow. Image analysis methods are already available to visualize the displacements of individual sand particles [86,87]. Simultaneous capturing of particle kinematics and the interstitial fluid flow field can potentially produce high-fidelity measurements of coupled flow fields that can be used for the development of numerical models of coupled flow. This will likely require the use of multiple cameras and image frames to capture phenomena at different scales but is indeed possible at this time. 

## 6. Conclusions

This study presented a methodology for exploring interstitial fluid flow using synthetic porous media in a refractive index matched model. The procedure involves the use of up-scaled extruded natural particles whose shape was obtained using dynamic image analysis of naturally occurring sand. The upscaled particles were made of a transparent hydrogel that were cast in 3D printed molds. The pore fluid was seeded with highly reflective silver-coated hollow micro-spheres in order to capture the fluid flow field. The method permitted capturing of interstitial fluid flow for PIV analysis in a 2D model, with the aid of a laser and a highspeed camera. The technique was applied to explore the role of particle roundness on flow. Comparison of steady state flow between scaled up particles of Ottawa sand and perfect cylinders having the same porosity and size permitted the following observations:

- Seepage velocity in each pore is different for both Cylindrical particles and Ottawa sands in a uniform geometry. The maximum and standard deviation of velocity in Ottawa Sand exhibit a larger difference. These variations in seepage velocity reflect turbulence which likely resulted from the effects of irregular particle shape in Ottawa Sand.
- The interstitial flow is more turbulent in Ottawa sand compared to cylindrical particles. Angular sands are expected to experience far more turbulence.
- The observed hydraulic gradient is not linear proportional to the discharge velocity and seepage velocity. Particle shape affect the hydraulic conductivity even though in the same void ratio and porosity.
- Smooth curvature of Cylindrical particles and symmetrical geometry is prone to causing small turbulence, while turbulence between Ottawa particles is larger in magnitude. Magnitude of turbulence is related to particle roundness.
- Flow between Ottawa sand is more concentrated in the downward vertical direction than for the Cylindrical particles. Cylindrical particles are perfectly placed, resulting in identical interstitial flow paths while the Ottawa particles are randomly spaced resulting in somewhat arbitrarily sized interstitial flow paths. Water follows the path of least resistance and small differences in the flow channels resulted in a more concentrated downward flow in the Ottawa particles than for the cylindrical ones, similar to the Mathew effect.
- These observations may lead to phenomena such as piping and internal erosion under steady-state and quasi steady-state flow, and have profound implications for a variety of geotechnical, geo-environmental and industrial applications such as filtration, environmental remediation, and resource recovery.

## Figures and Tables

**Table 1:** Average seepage velocity parameters.

	Cylindrical Particles	Ottawa Sand
Discharge velocity *v*	0.024 cm/s	0.058 cm/s
Porosity *n*	0.37	0.37
Hydraulic gradient *i*	4.6	5.7
Hydraulic Conductivity (observed) *k = v/i*	0.005 cm/s	0.01 cm/s
Seepage velocity *Theoretical v_s_ = v/n*	0.06 cm/s	0.16 cm/s
Seepage velocity *Observed* via *PIV*	0.04 cm/s	0.2 cm/s
Tortuosity *T*	0.63	0.88
Interstitial velocity [84] *v_i_* = *v*/*nT*	0.10 cm/s	0.19 cm/s

## Data Availability

Inter-particle fluid flow images employed in this study are available from the corresponding author upon reasonable request.

## Nomenclature

*C_k_*	Kozeny constant
*C_s_*	Shape constant of cross section
*De*	Superficial Effective Diameter
*D_h_*	Hydraulic diameter: *D_h_* = 4*n/S_s_*
*D_m_*	Grain diameter
ΔP	Pressure difference
*e*	Void ratio: Ratio of volume of voids to the volume of solids
*i*	Hydraulic gradient *i*: $\frac{\Delta P}{L}$
*k*	Hydraulic conductivity
*L*	Porous medium length
*L_e_*	Length to the actual pore flow path
*µ*	Fluid viscosity
*n*	Porosity: Ratio of volume of voids to the total volume
*R_h_*	Hydraulic radius: Flow area divided by wetted perimeter: *R_h_ = D_h_*/4
*S_s_*	Specific surface area
*T*	Tortuosity (Hydraulic tortuosity): *T* = *L/L_e_*
*v**	Average interstitial flow velocity
*v*	Discharge velocity: Apparent flow velocity through a medium
*v_s_*	Seepage velocity: *v_s_* = *v/n*
*v_i_*	Interstitial velocity: *v_i_*: *v/nT*
